# 

**Published:** 1908-06

**Authors:** 


### BOOKS RECEIVED.

Index to the Transactions of the American Laryngological, Rhinological and Otological Society. From 1896 to 1907 inclusive. Published by the Society. 1908.

The Sexual Question. A Scientific, Psychological, Hygienic and Sociological Study for the cultured classes. By August Forel, M.D., Ph.D., LL.D., formerly Professor of Psychiatry at and Director of the Insane Asylum in Zurich, Switzerland. English adaption by C. F. Marshall, M.D., F.R.C.S., late Assistant Surgeon to the Hospital for Diseaes of the Skin, London. Octavo, pp. 536. Illustrated. New York: Rebman Company.

Righthandedness and lefthandedness, with Chapters treating of the writing posture, the rule of the road, etc. By George M. Gould, M.D. Duodecimo, pp. 210. Illustrated. Philadelphia and London: J. B. Lippincott Co. (Cloth, $1.25.)

Transactions of the American Urological Association. Sixth annual meeting held at Atlantic City, June 3, and 4, 1907. Edited by Charles Greene Cumston, M.D.

Bureau of the Census. S. N. D. North, Director. Mortality Statistics for 1906. Seventh annual report. Washington: Government Printing Office. 1908.

Diseases of the Nose, Throat and Ear. Medical and Surgical. By William Lincoln Ballenger, M.D., Professor of Otology, Rhinology and Laryngology, College of Physicians and Surgeons, of Chicago University of Illinois. Octavo, 896 pages, with 467 engravings and 16 plates. Lea & Febiger, Publishers, Philadelphia and New York, 1908. (Cloth, $5.50 net.)

Concerning Lafcadio Hearn. By George M. Gould, M.D. With a Bibliography by Laura Stedman. Duodecimo, pp. 416. Illustrated. Philadelphia: George W. Jacobs & Co. (Cloth, $1.50.)

The Practical Medicine Series. Ten volumes. Issued under the general editorial charge of Gustavus P. Head, M.D., Professor of Laryngology and Rhinology in the Chicago Post-Graduate Medical School. Vols. 1, and 2, General Medicine, and Surgery. Series 1908. Chicago: The Year Book Publishers. (Prices, $1.50, $2.00; entire series, $10.00.)

The Development of Ophthalmology in America, 1800-1870. A Contribution to Ophthalmic History and Biography. An Address delivered in Abstract before the Section of Ophthalmology of the American Medical Association, June 4, 1907. Revised and enlarged. By Alvin A. Hubbell, M.D., Ph.D. Professor of Clinical Ophthalmology in the University of Buffalo, etc. Duodecimo, pp. 197. Illustrated by selected portraits and cuts. Chicago: W. T. Keener & Company. (Cloth, $1.75 net.)

